# Modeling the Correlation between *Z* and *B* in an X-ray Crystal Structure Refinement

**DOI:** 10.1101/2023.07.04.547724

**Published:** 2023-07-04

**Authors:** Trixia M. Buscagan, Douglas C. Rees

**Affiliations:** a Division of Chemistry and Chemical Engineering 147-75, Howard Hughes Medical Institute, California Institute of Technology, 1200 E. California Blvd., Pasadena, CA 91125 USA

**Keywords:** occupancy refinement, temperature factor refinement, iron-sulfur clusters, metalloproteins

## Abstract

We have examined how the refined *B*-factor changes as a function of *Z* (the atomic number of a scatterer) at the sulfur site of the [4Fe:4S] cluster of the nitrogenase iron protein by refinement. A simple model is developed that quantitatively captures the observed relationship between *Z* and *B*, based on a Gaussian electron density distribution with a constant electron density at the position of the scatterer. From this analysis, the fractional changes in *B* and *Z* are found to be similar. The utility of *B*-factor refinement to potentially distinguish atom types reflects the *Z* dependence of X-ray atomic scattering factors; the weaker dependence of electron atomic scattering factors on *Z* implies that distinctions between refined values of *B* in an electron scattering structure will be less sensitive to the atomic identity of a scatterer than for the case with X-ray-diffraction. This behavior provides an example of the complementary information that can be extracted from different types of scattering studies.

## Introduction

We recently reported a series of selenium-incorporated nitrogenase iron (Fe) protein crystal structures in which mixed occupancies of sulfur and selenium were observed at the chalcogenide sites of the [4Fe:4X] cluster (X = S, Se (Buscagan *et al*., 2022)). Occupancy refinements of these sites were correlated with shifts in the *B*-factor, reflecting the well-recognized correlation between occupancy and *B*-factor parameters that is most frequently encountered with solvents during protein structure refinement (Watenpaugh *et al*., 1978, Kundrot & Richards, 1987, Bhat, 1989, Jensen, 1990). By fixing the *B*-factors of the X atoms to match the *B*-factors of the Fe atoms, consistent with observations on other metalloprotein systems (Wittenborn *et al*., 2018, Jeoung *et al*., 2022), we refined occupancies at the mixed chalcogenide sites with minimal residual density in the *F*_obs_ - *F*_calc_ difference maps.

From that study, we became interested in characterizing the correlation between the atomic number, *Z* (as a proxy for occupancy), and the refined *B*-factor, to address two questions:

- for a given change in *Z*, how much does *B* change?
- can a physical model be devised that captures this relationship?

**Figure 1:**
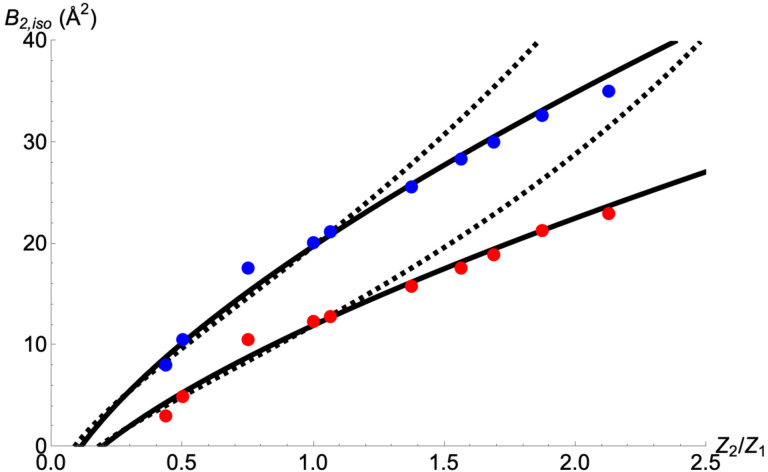
Refined *B*_2,iso_ values as a function of *Z*_2_/*Z*_1_, the ratio of the atomic number of the scatterer refined in the chalcogenide site (*Z*_2_) relative to *Z*_1_ = 16 (the true scatterer, sulfur), in the [4Fe:4S] cluster of the nitrogenase Fe protein (PDB data sets 7TPW (red circles) and 7TPY (blue circles)). The solid and dashed lines represent the fits to Eq. 4 and Eq. 6, respectively, with *Z*_1_ = 16 e^−^ and *B*_0_ = 6 Å^2^ for both structures, and *B*_1,iso_ = 12.0 Å^2^ and 19.8 Å^2^ for the 7TPW and 7TPY structures, respectively.

To address the first question, the sulfide ligands in the [4Fe:4S] clusters of nitrogenase Fe protein PDB data sets 7TPW and 7TPY (with resolutions of 1.18 Å and 1.48 Å, respectively) were modeled with different elements in place of sulfur, and the *B*-factors refined. In these structures, the sulfurs in the cluster were fully occupied; i.e., no selenium was present. PHENIX (Liebschner *et al*., 2019), and COOT (Emsley *et al*., 2010) were used for this analysis, and the cluster coordinates were fixed so that only the *B*-factors were refined. Neutral scattering factors were used for the cluster atoms. The refined *B* values are provided in Appendix A. Over a *Z* range from 7 (N) to 34 (Se), the expected positive correlations between *Z* and refined *B* values are evident for both structures (Figure 1). A linear fit to this data for 8 ≤ *Z* ≤ 25 indicates that over this range, *B* varies approximately linearly with changes in *Z*; the fractional change in *B* relative to the fractional change in *Z* was found to be approximately 0.9 and 0.8 for the 7TPW and 7TPY data sets, respectively (Appendix A). Thus, the answer to the first question is that for this system, a 10% change in *Z* results in an approximately 8–9% change in *B*.

## Modeling the relationship between Z and B

To model the relationship between *Z* and *B*, we represent a scatterer by a single Gaussian with atomic number *Z* and overall temperature factor *B* (Teneyck, 1977). The electron density *ρ*(*r*) is then described:

Eq. 1 $$\rho (r)=Z{\left(\frac{4\pi }{B}\right)}^{3/2}e^{-4\pi^{2}r^{2}/B}$$

with

Eq. 2 $$\rho (0)=Z{\left(\frac{4\pi }{B}\right)}^{3/2}$$

It is important to recognize that the *B* in Eq. 1 and Eq. 2 includes contributions from both the atomic scattering factor *B*_0_ and the isothermal temperature factor *B*_iso_, with *B* = *B*_0_ + *B*_iso_. From Eq. 2 and *ρ*(0) calculated with the Cromers and Mann atomic scattering factors (Cromer & Mann, 1968) and *B*_iso_ = 16 Å^2^, *B*_0_ is found to be approximately 8 Å^2^ and 6 Å^2^ for N and S, respectively. If the true *Z/B* for a given atom are *Z*_1_ and *B*_1_, but the refinement is conducted with *Z*_2_, the corresponding *B*_2_ will be shifted from the true value to compensate for the incorrect occupancy. We developed two simple models to capture the possible relationship between *Z*_*2*_ and *B*_*2*_:

**Model 1:***B*_2_ is calculated for a given *Z*_2_ such that the density at the atomic position, *ρ*(0), has the same value as for *Z*_1_, *B*_1_. For a single Gaussian, this is equivalent to equating *ρ*(0) in Eq. 2 calculated for either *Z*_1_, *B*_1_ or *Z*_2_, *B*_2_, which gives
Eq. 3 $$\frac{Z_{1}}{B_{1}^{3/2}}=\frac{Z_{2}}{B_{2}^{3/2}}\Rightarrow B_{2}=B_{1}{\left(\frac{Z_{2}}{Z_{1}}\right)}^{2/3}$$
Eq. 4 $$B_{2,iso}=\left(B_{1,iso}+B_{0}\right){\left(\frac{Z_{2}}{Z_{1}}\right)}^{2/3}-B_{0}$$
The ratio *Z*_2_*/Z*_1_ corresponds to the occupancy of the *Z*_1_ scatterer at the site (to within the approximation that the shape of the atomic scattering factor is independent of *Z*).
**Model 2:**In this case, *B*_2_ is calculated for a given *Z*_2_ to minimize the square of the difference density over the atomic volume:
Eq. 5 $$\Delta \rho^{2}=\int_{0}^{\infty }{\left(\rho_{1}(r)-\rho_{2}(r)\right)}^{2}4\pi r^{2}dr\;=\int_{0}^{\infty }{\left(\left[Z_{1}{\left(\frac{4\pi }{B_{1}}\right)}^{3/2}e^{-4\pi^{2}r^{2}/B_{1}}\right]-\left[Z_{2}{\left(\frac{4\pi }{B_{2}}\right)}^{3/2}e^{-4\pi^{2}r^{2}/B_{2}}\right]\right)}^{2}4\pi r^{2}dr$$
  From the condition that $\frac{\partial \Delta \rho^{2}}{\partial B_{2}}=0$ at the minimum, one can derive (Appendix B)
Eq. 6 $$B_{2}=B_{1}\frac{{\left(\frac{Z_{2}}{Z_{1}}\right)}^{2/5}}{2-{\left(\frac{Z_{2}}{Z_{1}}\right)}^{2/5}}\Rightarrow B_{2,iso}=\left(B_{1,iso}+B_{0}\right)\frac{{\left(\frac{Z_{2}}{Z_{1}}\right)}^{2/5}}{2-{\left(\frac{Z_{2}}{Z_{1}}\right)}^{2/5}}-B_{0}$$
The variations in *B*_2,iso_ as a function of *Z*_2_/*Z*_1_ were evaluated from Eq. 4 and Eq. 6 (Figure 1). For these calculations, *Z*_1_ = 16 e^−^ and *B*_0_ = 6 Å^2^, with *B*_1,iso_ = 12.0 Å^2^ and 19.8 Å^2^ for the 7TPW and 7TPY structures, respectively. (These *B*_1,iso_ values correspond to the average *B-*factor for the two Fe sites in each structure; Appendix A.) As illustrated in Figure 1, while both Eq. 4 and Eq. 6 fit the refined *B* values reasonably well for *Z*_2_/*Z*_1_ < 1, the fit of Eq. 4 is superior over the entire range tested. This was a surprising result to us, as we anticipated that the Δρ^2^ model would better capture the structure refinement process; instead, the isolated atom approximation (reflected in the upper limit of r = ∝ in Eq. 5) for a macromolecular structure refinement is evidently less accurate relative to the localized treatment implicit in the derivation of Eq. 4.

## Discussion

We recognize that the approximations used to derive the relationship between *Z* and *B* in Eq. 4 are inferior to the results of a full structure refinement. Nevertheless, Eq. 4 can provide a useful starting point to evaluate the relationship between *Z* and *B* for scatterers involving either species of unknown atomic identity (such as the original analysis of the interstitial ligand in the nitrogenase FeMo-cofactor (Einsle *et al*., 2002), that prompted the initial development of model 2); partial occupancy (such as solvents (Watenpaugh *et al*., 1978)); mixed atomic composition (exchange reactions or disorder, as in (Spatzal *et al*., 2015, Buscagan *et al*., 2022)); or incorrect modeling of residues adopting distinct flipped orientations, such as the side chains of asparagine and glutamine residues where flipping interchanges N and O atoms (Word *et al*., 1999, Weichenberger & Sippl, 2006).

The utility of *B*-factor refinement to potentially distinguish atom types reflects the *Z* dependence of X-ray atomic scattering factors. An instructive comparison may be drawn to electron atomic scattering factors that depend on the Coulomb potential of the scatterer and have a less significant dependence on *Z*. In particular, *ρ*(0)for fully occupied C, N and O atoms calculated using electron scattering factors (parameterized in (Saha *et al*., 2021)) with a *B* = 16 Å^2^ vary by less than 2%, while the corresponding variation between the C and O atoms using X-ray scattering factors is approximately 50%. Consequently, the distinctions between the C, N, and O atoms in an electron scattering map are less evident than in X-ray scattering maps. This property of electron scattering was reflected in our recent structure determination of an antibiotic peptide by micro-electron diffraction, where the orientation of a histidine sidechain in a novel cross link could not be established from an analysis of the *B-*factors for the two distinct rotamers (Miller *et al*., 2022). A “multi-messenger” approach using combinations of X-ray (with anomalous scattering, if applicable), electron, and neutron diffraction can provide additional experimental restraints to help resolve ambiguities arising in structure refinements from the correlation between *Z* and/or occupancy with the *B*-factor of scatterers.

## Appendix A: *B*-factor refinement of different scatterers in the sulfur sites of a [4Fe:4S] cluster

**Table:**

7TPW refinement										
X = element	N	O	Mg	S	Cl	Ti	Mn	Co	Zn	Se
atomic number *(Z)*	7	8	12	16	17	22	25	27	30	34
Z_2_/Z_1_	0.44	0.50	0.75	1.00	1.06	1.38	1.56	1.69	1.88	2.13
cluster atom				refined *B* values (Å^2^)						
Fe1 (Å^2^)	11.82	11.90	11.99	12.02	12.11	12.07	12.12	12.28	12.27	12.30
Fe2 (Å^2^)	11.86	11.88	12.01	11.98	12.03	12.06	12.03	12.14	12.29	12.18
X3 (Å^2^)	3.41	5.38	10.85	12.75	13.30	16.23	18.18	19.42	21.87	23.77
X4 (Å^2^)	2.80	4.61	10.22	11.99	12.49	15.41	17.18	18.53	20.84	22.21
X *B*_avg_ (Å^2^)	3.11	5.00	10.54	12.37	12.90	15.82	17.68	18.98	21.36	22.99
R_work_	14.20	14.16	14.11	14.07	14.09	14.12	14.14	14.17	14.24	14.24
R_free_	16.09	16.03	15.92	15.89	15.84	16.06	16.04	16.04	16.34	16.34
				7TPY refinement						
X = element	N	O	Mg	S	Cl	Ti	Mn	Co	Zn	Se
cluster atom				refined *B* values (Å^2^)						
Fe1 (Å^2^)	19.82	19.73	19.91	19.73	19.91	19.77	19.60	19.60	19.44	19.38
Fe2 (Å^2^)	19.84	20.01	20.13	19.83	20.02	20.02	19.75	19.75	19.76	19.59
X3 (Å^2^)	8.33	10.86	17.83	20.38	21.47	26.02	28.68	30.40	33.01	35.31
X4 (Å^2^)	7.90	10.39	17.38	19.81	20.96	25.40	28.03	29.80	32.30	34.76
X *B*_avg_ (Å^2^)	8.12	10.63	17.61	20.10	21.22	25.71	28.36	30.10	32.66	35.04
R_work_	17.34	17.25	17.18	17.17	17.23	17.24	17.26	17.23	17.27	17.36
R_free_	19.21	18.98	18.85	19.01	18.92	19.02	19.03	19.00	19.15	19.06

The *B*-factors are refined as a function of the element in the chalcogenide site of the [4Fe:4S] cluster for two nitrogenase Fe protein structures (PDB IDs 7TPW and 7TPY, with resolutions of 1.18 Å and 1.48 Å, respectively)) detailed in (Buscagan *et al*., 2022). The cluster sits on a crystallographic two-fold axis, so that the crystallographically unique sites are designated Fe1, Fe2, X3 and X4. Least squares lines fit to the *B*_avg_ values of the scatterers at the X position over the range 8 ≤ *Z* ≤ 25 yielded slopes of 0.692 and 0.987 for the 7TPW and 7TPY data sets, respectively; when normalized to the appropriate *Z* and *B* values for S, the fractional changes in *B* with a fractional change in *Z* are found to be 0.896 and 0.786, respectively.

## Appendix B: Derivation of Equation 6

Eq. 6 can be derived from Eq. 5 as follows. The integral and derivative were evaluated with Mathematica^®^.

$$\Delta \rho^{2}=\int_{0}^{\infty }{\left(\rho_{1}(r)-\rho_{2}(r)\right)}^{2}4\pi r^{2}dr\;=\int_{0}^{\infty }{\left(\left[Z_{1}{\left(\frac{4\pi }{B_{1}}\right)}^{3/2}e^{-4\pi^{2}r^{2}/B_{1}}\right]-\left[Z_{2}{\left(\frac{4\pi }{B_{2}}\right)}^{3/2}e^{-4\pi^{2}r^{2}/B_{2}}\right]\right)}^{2}4\pi r^{2}dr\;=2\pi^{3/2}\left[\frac{\sqrt{2}Z_{1}^{2}}{B_{1}^{3/2}}-\frac{8Z_{1}Z_{2}}{{\left(B_{1}+B_{2}\right)}^{3/2}}+\frac{\sqrt{2}Z_{2}^{2}}{B_{2}^{3/2}}\right]\;\frac{\partial \Delta \rho^{2}}{\partial B_{2}}=2\pi^{3/2}Z_{2}\left(\frac{12Z_{1}}{{\left(B_{1}+B_{2}\right)}^{5/2}}-\frac{3Z_{2}}{\sqrt{2}B_{2}^{5/2}}\right)=0\;\frac{B_{2}^{5/2}}{{\left(B_{1}+B_{2}\right)}^{5/2}}=\frac{3Z_{2}}{12\sqrt{2}Z_{1}}=\frac{1}{{\left(\sqrt{2}\right)}^{5/2}}\frac{Z_{2}}{Z_{1}}\;\frac{{\left(\sqrt{2}\right)}^{5/2}B_{2}^{5/2}}{{\left(B_{1}+B_{2}\right)}^{5/2}}=\frac{Z_{2}}{Z_{1}}\;\frac{2B_{2}}{\left(B_{1}+B_{2}\right)}={\left(\frac{Z_{2}}{Z_{1}}\right)}^{2/5}\;2B_{2}={\left(\frac{Z_{2}}{Z_{1}}\right)}^{2/5}\left(B_{1}+B_{2}\right)\;B_{2}\left(2-{\left(\frac{Z_{2}}{Z_{1}}\right)}^{2/5}\right)=B_{1}{\left(\frac{Z_{2}}{Z_{1}}\right)}^{2/5}\;B_{2}=B_{1}\frac{{\left(\frac{Z_{2}}{Z_{1}}\right)}^{2/5}}{\left(2-{\left(\frac{Z_{2}}{Z_{1}}\right)}^{2/5}\right)}$$
